# Supplementary material for: Quality adjustment and analysis of human resource prices in China: Based on a hedonic price model
Source: PLoS One. 2024 Apr 2;19(4):e0297352. doi: 10.1371/journal.pone.0297352 (PMC10986967; doi:10.1371/journal.pone.0297352)
Supplement: S1 File — (DOCX) [file pone.0297352.s001.docx]

**Table 1: HR prices in 31 provinces of China (all the years) (yuan/person)**

| **Province** | **1995** | **1996** | **1997** | **1998** | **1999** | **2000** | **2001** | **2002** | **2003** |
| --- | --- | --- | --- | --- | --- | --- | --- | --- | --- |
| Shanghai | 6278 | 7336 | 7881 | 9228 | 10253 | 10746 | 10761 | 10560 | 11576 |
| Beijing | 7740 | 9272 | 9111 | 10174 | 11392 | 13683 | 15570 | 16355 | 18129 |
| Guangdong | 7243 | 7037 | 7801 | 8088 | 8166 | 10167 | 9256 | 12580 | 13854 |
| Tianjin | 3096 | 3640 | 3923 | 4712 | 5260 | 5911 | 6782 | 8227 | 8755 |
| Jiangsu | 2761 | 3518 | 3967 | 4245 | 4811 | 5657 | 6132 | 7492 | 8234 |
| Zhejiang | 4723 | 5401 | 5977 | 6443 | 6860 | 7672 | 8558 | 9348 | 10208 |
| Fujian | 3475 | 4102 | 4859 | 5123 | 5481 | 6222 | 6906 | 7600 | 8191 |
| Liaoning | 3074 | 3491 | 3716 | 4151 | 4247 | 4510 | 4883 | 6178 | 6739 |
| Neimenggu | 2068 | 2264 | 2478 | 3196 | 3636 | 4032 | 4636 | 5144 | 5996 |
| Shanxi | 2119 | 2322 | 2604 | 2720 | 2953 | 3522 | 4001 | 5411 | 6077 |
| Shandong | 1737 | 2225 | 2734 | 2894 | 3051 | 3259 | 3721 | 4101 | 4962 |
| Chongqing | 2001 | 2310 | 2429 | 2699 | 2845 | 3193 | 3528 | 4486 | 5746 |
| Xijiang | 4052 | 4342 | 4397 | 4856 | 5189 | 3916 | 4682 | 5463 | 5982 |
| Hebei | 2134 | 2486 | 2864 | 2912 | 3192 | 3417 | 3467 | 4066 | 4610 |
| Jinlin | 1918 | 2841 | 3516 | 3671 | 3998 | 4252 | 4766 | 5856 | 7001 |
| Ningxia | 1640 | 1755 | 1961 | 2331 | 2448 | 3049 | 3516 | 3736 | 4189 |
| Jiangxi | 1706 | 1908 | 2122 | 2331 | 2702 | 3093 | 3573 | 4181 | 4733 |
| Shanxi | 1865 | 2474 | 3398 | 3740 | 4313 | 4934 | 2999 | 3583 | 4066 |
| Heilongjiang | 3489 | 3785 | 3664 | 3787 | 4171 | 4377 | 4684 | 5369 | 6023 |
| Hainan | 1069 | 1768 | 1806 | 1981 | 1994 | 2487 | 2856 | 3323 | 3387 |
| Hunan | 2151 | 2454 | 2653 | 2931 | 3205 | 3721 | 3973 | 4278 | 4801 |
| Qinghai | 1694 | 2023 | 2227 | 2151 | 2108 | 2482 | 3060 | 3239 | 3607 |
| Hubei | 1837 | 2000 | 2118 | 2524 | 2742 | 2967 | 3380 | 4239 | 4687 |
| Guangxi | 2592 | 4728 | 5214 | 5804 | 6170 | 5769 | 6651 | 8065 | 3962 |
| Anhui | 1446 | 1929 | 2005 | 2152 | 2465 | 2558 | 2850 | 3345 | 3821 |
| Sichuan | 1543 | 1809 | 2232 | 2469 | 2748 | 2775 | 2902 | 3118 | 3515 |
| Guizhou | 3876 | 4034 | 4727 | 4696 | 5341 | 2036 | 2127 | 2242 | 2610 |
| Henan | 1108 | 1356 | 1634 | 1723 | 1872 | 1879 | 2119 | 2659 | 3068 |
| Gansu | 1043 | 1165 | 1219 | 1428 | 1849 | 2103 | 2307 | 2755 | 3097 |
| Xizang | 1699 | 2039 | 1967 | 2133 | 2462 | 4179 | 4898 | 5514 | 6692 |
| Yunnan | 1197 | 1455 | 1643 | 1808 | 1948 | 2160 | 2465 | 3014 | 3330 |

**Table 1 continued1**

| **Province** | **2004** | **1996** | **1997** | **1998** | **1999** | **2000** | **2001** | **2002** | **2012** |
| --- | --- | --- | --- | --- | --- | --- | --- | --- | --- |
| Shanghai | 11516 | 29581 | 34710 | 41613 | 48492 | 51178 | 59118 | 60290 | 62600 |
| Beijing | 17434 | 21315 | 25281 | 27560 | 30471 | 35472 | 39946 | 43426 | 47851 |
| Guangdong | 15053 | 15467 | 17165 | 18873 | 19842 | 21746 | 25136 | 28176 | 32222 |
| Tianjin | 9595 | 13097 | 14896 | 16625 | 19707 | 22115 | 25732 | 29206 | 33448 |
| Jiangsu | 9143 | 10997 | 12102 | 13542 | 15161 | 16147 | 18137 | 21989 | 25247 |
| Zhejiang | 11061 | 12644 | 13345 | 14282 | 15398 | 16294 | 18457 | 20017 | 21984 |
| Fujian | 8658 | 9857 | 11233 | 12226 | 13820 | 15269 | 17410 | 17784 | 20009 |
| Liaoning | 7138 | 8712 | 9602 | 11544 | 13426 | 14227 | 15628 | 17661 | 19905 |
| Neimenggu | 6824 | 7879 | 9068 | 11277 | 12663 | 13852 | 15572 | 17751 | 19777 |
| Shanxi | 7030 | 8348 | 9360 | 10593 | 10793 | 11669 | 13473 | 16476 | 18615 |
| Shandong | 5568 | 7684 | 8925 | 10107 | 11181 | 12109 | 13937 | 16054 | 18465 |
| Chongqing | 6790 | 7874 | 9543 | 11450 | 13149 | 14400 | 14411 | 16013 | 18049 |
| Xijiang | 6164 | 6728 | 7839 | 9754 | 10476 | 11335 | 13302 | 14493 | 15931 |
| Hebei | 5259 | 6139 | 7006 | 8335 | 9145 | 10325 | 11492 | 13142 | 14783 |
| Jinlin | 7447 | 8260 | 9403 | 10944 | 12003 | 12632 | 13561 | 15177 | 16492 |
| Ningxia | 4860 | 5661 | 6336 | 7794 | 9468 | 9972 | 11869 | 13698 | 15498 |
| Jiangxi | 5083 | 5824 | 6639 | 7962 | 8869 | 9564 | 10473 | 12320 | 14443 |
| Shanxi | 4488 | 5301 | 6032 | 7337 | 8831 | 9939 | 11647 | 14374 | 16642 |
| Heilongjiang | 6474 | 6828 | 7563 | 8580 | 9302 | 10377 | 11130 | 12471 | 14070 |
| Hainan | 5997 | 6548 | 7527 | 8891 | 9915 | 10697 | 12044 | 14317 | 16051 |
| Hunan | 5566 | 5479 | 6196 | 7370 | 8117 | 8972 | 10186 | 11510 | 13590 |
| Qinghai | 4028 | 4636 | 5185 | 6686 | 7525 | 8362 | 9524 | 11457 | 12962 |
| Hubei | 4954 | 5451 | 6376 | 7495 | 8292 | 9126 | 10679 | 12299 | 14226 |
| Guangxi | 4522 | 4534 | 4833 | 6020 | 6961 | 7614 | 7954 | 9075 | 12964 |
| Anhui | 4435 | 5151 | 6092 | 13168 | 8205 | 9060 | 9566 | 11115 | 12962 |
| Sichuan | 3914 | 4482 | 5356 | 6574 | 7596 | 8669 | 9926 | 11522 | 13377 |
| Guizhou | 2983 | 3119 | 3709 | 4363 | 5576 | 6364 | 7799 | 9165 | 10707 |
| Henan | 3608 | 4076 | 4793 | 5836 | 6815 | 7570 | 8336 | 9670 | 11248 |
| Gansu | 3648 | 4313 | 4759 | 5604 | 5962 | 6608 | 7404 | 8790 | 10274 |
| Xizang | 8082 | 5192 | 3976 | 5123 | 6001 | 6446 | 6931 | 7094 | 7805 |
| Yunnan | 3602 | 3736 | 4327 | 5069 | 5597 | 6351 | 7272 | 8571 | 10563 |

**Table 1 continued2**

| **Province** | 2013 | 2014 | 2015 | 2016 | 2017 | 2018 | 2019 | 2020 | Mean |
| --- | --- | --- | --- | --- | --- | --- | --- | --- | --- |
| Shanghai | 58694 | 54911 | 55002 | 57978 | 58158 | 60406 | 69000 | 77594 | 37518 |
| Beijing | 49389 | 52252 | 55386 | 59000 | 61334 | 65608 | 73062 | 80516 | 34490 |
| Guangdong | 33777 | 32128 | 34997 | 37812 | 41190 | 44007 | 50230 | 56454 | 23403 |
| Tianjin | 27277 | 29791 | 33325 | 36872 | 40413 | 43849 | 53422 | 62994 | 21641 |
| Jiangsu | 27394 | 26763 | 29541 | 32318 | 35531 | 38455 | 44623 | 50790 | 18258 |
| Zhejiang | 22732 | 23311 | 23915 | 25303 | 26975 | 28751 | 43230 | 57708 | 17715 |
| Fujian | 20582 | 20153 | 21191 | 22988 | 25214 | 28024 | 39046 | 50069 | 15596 |
| Liaoning | 20461 | 21123 | 23785 | 26624 | 28225 | 30558 | 35601 | 40643 | 14840 |
| Neimenggu | 19360 | 18885 | 21337 | 22919 | 23974 | 29487 | 36202 | 42918 | 13969 |
| Shanxi | 19616 | 20595 | 22162 | 26281 | 27479 | 28771 | 32287 | 35802 | 13503 |
| Shandong | 18902 | 18520 | 20535 | 22671 | 25297 | 29037 | 36758 | 44480 | 13420 |
| Chongqing | 19496 | 18059 | 19631 | 21566 | 23905 | 26869 | 33747 | 40626 | 13262 |
| Xijiang | 16715 | 17510 | 20242 | 21567 | 23098 | 24343 | 30104 | 35866 | 12629 |
| Hebei | 16587 | 17807 | 20100 | 22174 | 24672 | 27175 | 34686 | 42196 | 12314 |
| Jinlin | 15488 | 15509 | 16448 | 17877 | 19968 | 23072 | 29753 | 36435 | 12242 |
| Ningxia | 17505 | 18338 | 19365 | 20396 | 23804 | 26070 | 34659 | 43248 | 12045 |
| Jiangxi | 16316 | 17109 | 18878 | 20812 | 23331 | 25934 | 34335 | 42735 | 11807 |
| Shanxi | 17103 | 17121 | 18541 | 20396 | 22447 | 24599 | 29833 | 35067 | 11580 |
| Heilongjiang | 14960 | 16387 | 17749 | 18500 | 19894 | 21350 | 29049 | 36748 | 11569 |
| Hainan | 16377 | 16776 | 18677 | 21007 | 22213 | 23325 | 31030 | 38735 | 11569 |
| Hunan | 14660 | 15491 | 17570 | 19924 | 22776 | 25380 | 32985 | 40590 | 11405 |
| Qinghai | 14657 | 15854 | 17596 | 19873 | 22283 | 24208 | 32375 | 40541 | 10782 |
| Hubei | 14924 | 14789 | 16703 | 18276 | 20290 | 22140 | 27519 | 32899 | 10497 |
| Guangxi | 13405 | 13015 | 14431 | 16022 | 18008 | 18886 | 26862 | 34839 | 10342 |
| Anhui | 13821 | 13628 | 14966 | 16169 | 17564 | 19846 | 29419 | 38991 | 10259 |
| Sichuan | 14057 | 13977 | 15371 | 16796 | 18340 | 20521 | 27325 | 34128 | 9809 |
| Guizhou | 12174 | 12444 | 13836 | 15360 | 16952 | 18558 | 25934 | 33310 | 9002 |
| Henan | 12063 | 12078 | 13286 | 13964 | 15286 | 17094 | 26922 | 36749 | 8724 |
| Gansu | 11390 | 11694 | 13004 | 14597 | 16534 | 18358 | 25061 | 31763 | 8336 |
| Xizang | 8022 | 8413 | 9737 | 10637 | 11506 | 13265 | 25935 | 38606 | 8244 |
| Yunnan | 10755 | 10817 | 12350 | 13369 | 15065 | 17209 | 23096 | 28983 | 7914 |

**Table 4: Regression coefficients for variables in each year**

| **year** | **C** | **lnX1** | **lnX2** | **lnX3** | **lnX4** | **lnX5** | **lnX6** | **lnX7** | **lnX8** | **lnX9** | **lnX10** |
| --- | --- | --- | --- | --- | --- | --- | --- | --- | --- | --- | --- |
| 1995 | 7.0314 | 0.1678 | 0.0315 | -0.0905 | 0.0942 | 0.067 | 0.1389 | 0.0572 | 0.0478 | 0.0472 | 0.0941 |
| 1996 | 7.0494 | 0.1694 | 0.0595 | -0.0751 | 0.0954 | 0.0701 | 0.1359 | 0.0482 | 0.04 | 0.0472 | 0.091 |
| 1997 | 7.1134 | 0.1992 | 0.0598 | -0.0717 | 0.0889 | 0.0608 | 0.1268 | 0.0503 | 0.0474 | 0.0433 | 0.0886 |
| 1998 | 7.107 | 0.1803 | 0.0497 | -0.0644 | 0.0954 | 0.071 | 0.1447 | 0.0524 | 0.0507 | 0.0475 | 0.0944 |
| 1999 | 7.1604 | 0.126 | 0.0365 | -0.0539 | 0.0999 | 0.0817 | 0.153 | 0.0439 | 0.0483 | 0.0508 | 0.0939 |
| 2000 | 6.9449 | 0.0853 | 0.1021 | -0.03 | 0.1152 | 0.1014 | 0.1767 | 0.05 | 0.0482 | 0.059 | 0.0932 |
| 2001 | 6.891 | 0.0815 | 0.0901 | -0.0128 | 0.1212 | 0.1065 | 0.1867 | 0.0441 | 0.0499 | 0.0625 | 0.0914 |
| 2002 | 6.8247 | 0.0952 | 0.0619 | -0.0112 | 0.1371 | 0.1223 | 0.2017 | 0.0354 | 0.0324 | 0.0639 | 0.0957 |
| 2003 | 6.821 | 0.0431 | 0.0347 | -0.0106 | 0.1601 | 0.1558 | 0.2143 | 0.0366 | 0.0379 | 0.0661 | 0.1008 |
| 2004 | 6.9209 | 0.0822 | 0.0336 | -0.0348 | 0.1602 | 0.1559 | 0.1805 | 0.0392 | 0.0366 | 0.054 | 0.1151 |
| 2005 | 6.8412 | 0.1607 | 0.0726 | -0.075 | 0.138 | 0.1437 | 0.2335 | 0.0521 | 0.0304 | 0.0648 | 0.1076 |
| 2006 | 6.6612 | 0.2178 | 0.0944 | -0.0991 | 0.1387 | 0.1036 | 0.2347 | 0.0573 | 0.0531 | 0.0565 | 0.1163 |
| 2007 | 6.8058 | 0.3844 | 0.1118 | -0.084 | 0.1354 | 0.0709 | 0.2213 | 0.0496 | 0.0575 | 0.0559 | 0.1214 |
| 2008 | 6.4849 | 0.3763 | 0.1027 | -0.0919 | 0.1511 | 0.0619 | 0.2451 | 0.0519 | 0.0524 | 0.0574 | 0.1149 |
| 2009 | 6.7518 | 0.4108 | 0.1164 | -0.0506 | 0.1734 | 0.0761 | 0.1744 | 0.0909 | 0.054 | 0.0396 | 0.1274 |
| 2010 | 6.0368 | 0.3047 | 0.1037 | -0.0652 | 0.194 | 0.0174 | 0.177 | 0.0311 | 0.0995 | 0.0626 | 0.1488 |
| 2011 | 6.1349 | 0.4722 | 0.1614 | -0.093 | 0.1583 | 0.0617 | 0.307 | 0.0497 | 0.0512 | 0.0654 | 0.1442 |
| 2012 | 6.1934 | 0.5402 | 0.1703 | -0.0942 | 0.1318 | 0.0488 | 0.3069 | 0.0528 | 0.0511 | 0.0622 | 0.1465 |
| 2013 | 6.3595 | 0.4216 | 0.1482 | -0.0765 | 0.1394 | 0.0721 | 0.2967 | 0.0466 | 0.0449 | 0.0637 | 0.1415 |
| 2014 | 6.4365 | 0.3127 | 0.1265 | -0.0586 | 0.1426 | 0.0826 | 0.3217 | 0.0467 | 0.0453 | 0.0629 | 0.1421 |
| 2015 | 6.3391 | 0.2873 | 0.1329 | -0.0587 | 0.1293 | 0.1034 | 0.3421 | 0.0503 | 0.0498 | 0.0636 | 0.1505 |
| 2016 | 6.5803 | 0.3701 | 0.1686 | -0.0601 | 0.099 | 0.0684 | 0.3274 | 0.048 | 0.048 | 0.0596 | 0.1406 |
| 2017 | 6.6972 | 0.3487 | 0.1366 | -0.0614 | 0.1049 | 0.0714 | 0.3149 | 0.0496 | 0.0495 | 0.0576 | 0.1416 |
| 2018 | 7.3858 | 0.4059 | 0.1358 | -0.0653 | 0.0589 | 0.0116 | 0.2995 | 0.0523 | 0.0543 | 0.0548 | 0.14 |
| 2019 | 7.5418 | 0.3821 | 0.1266 | -0.0540 | 0.0491 | 0.0088 | 0.2712 | 0.0648 | 0.0642 | 0.0484 | 0.1468 |
| 2020 | 7.5531 | 0.3789 | 0.1269 | -0.059 | 0.0501 | 0.0097 | 0.2864 | 0.0698 | 0.0712 | 0.0499 | 0.1498 |
| Standard  deviation | 0.4028 | 0.1414 | 0.0436 | 0.0252 | 0.0368 | 0.0404 | 0.0690 | 0.0116 | 0.0133 | 0.0076 | 0.0232 |
| Mean | 6.7949 | 0.2694 | 0.0998 | -0.0616 | 0.1216 | 0.0771 | 0.2315 | 0.0508 | 0.0506 | 0.0564 | 0.1207 |

**Table 5: Comparison of the average nominal and actual HR prices**

**in 31 provinces**

| **Province** | **Average price of nominal human resources (yuan/person)** | **Average price of actual human resources (yuan/person)** | **Ratio of nominal to actual price means** |
| --- | --- | --- | --- |
| Jiangsu | 18258 | 8606 | 2.12 |
| Shanxi | 11580 | 5472 | 2.12 |
| Chongqing | 13262 | 6341 | 2.09 |
| Beijing | 34490 | 16521 | 2.09 |
| Henan | 8724 | 4215 | 2.07 |
| Gansu | 8336 | 4044 | 2.06 |
| Tianjin | 21641 | 10695 | 2.02 |
| Qinghai | 10782 | 5337 | 2.02 |
| Guizhou | 9002 | 4476 | 2.01 |
| Ningxia | 12045 | 6023 | 2.00 |
| Sichuan | 9809 | 4915 | 2.00 |
| Anhui | 10259 | 5183 | 1.98 |
| Hunan | 11405 | 5779 | 1.97 |
| Shandong | 13420 | 6809 | 1.97 |
| Shanghai | 37518 | 19091 | 1.97 |
| Hubei | 10497 | 5354 | 1.96 |
| Zhejiang | 17715 | 9147 | 1.94 |
| Neimenggu | 13969 | 7216 | 1.94 |
| Xizang | 8244 | 4300 | 1.92 |
| Hainan | 11569 | 6055 | 1.91 |
| Shanxi | 13503 | 7071 | 1.91 |
| Xijiang | 12629 | 6675 | 1.89 |
| Yunnan | 7914 | 4256 | 1.86 |
| Jiangxi | 11807 | 6363 | 1.86 |
| Guangdong | 23403 | 12694 | 1.84 |
| Hebei | 12314 | 6689 | 1.84 |
| Liaoning | 14840 | 8091 | 1.83 |
| Heilongjiang | 11569 | 6319 | 1.83 |
| Jinlin | 12242 | 6782 | 1.81 |
| Fujian | 15596 | 8674 | 1.80 |
| Guangxi | 10342 | 5901 | 1.75 |

**Data of figure1**

| **year** | **Amount of HR in China  (ten thousand people)** | **year** | **Amount of HR in China  (ten thousand people)** |
| --- | --- | --- | --- |
| 1995 | 68065 | 2008 | 75564 |
| 1996 | 68950 | 2009 | 75828 |
| 1997 | 69820 | 2010 | 76105 |
| 1998 | 70637 | 2011 | 76196 |
| 1999 | 71394 | 2012 | 76254 |
| 2000 | 72085 | 2013 | 76301 |
| 2001 | 72797 | 2014 | 76349 |
| 2002 | 73280 | 2015 | 76320 |
| 2003 | 73736 | 2016 | 76245 |
| 2004 | 74264 | 2017 | 76058 |
| 2005 | 74647 | 2018 | 75782 |
| 2006 | 74978 | 2019 | 75447 |
| 2007 | 75321 | 2020 | 75064 |

**Data of figure 2**

| Province | Average number of HR  (10,000 persons) | Average growth  rate (%) | Province | Average number of HR  (10,000 persons) | Average growth  rate (%) |
| --- | --- | --- | --- | --- | --- |
| Shandong | 5946 | 0.23 | Guizhou | 1960 | 0.17 |
| Henan | 5790 | 0.32 | Heilongjiang | 1794 | -0.21 |
| Guangdong | 5350 | 2.77 | Shanxi | 1628 | 0.80 |
| Sichuan | 4742 | 0.11 | Chongqing | 1616 | -0.09 |
| Jiangsu | 4618 | 0.44 | Gansu | 1492 | -0.43 |
| Anhui | 3820 | 0.04 | Jinlin | 1301 | -0.03 |
| Hunan | 3765 | -0.22 | Neimenggu | 1185 | 0.76 |
| Hebei | 3736 | 0.49 | Shanghai | 1034 | 2.22 |
| Hubei | 3512 | 0.03 | Beijing | 933 | 2.26 |
| Zhejiang | 3286 | 1.56 | Xijiang | 922 | 2.82 |
| Guangxi | 2695 | 0.28 | Tianjin | 664 | 0.92 |
| Yunnan | 2605 | 1.07 | Hainan | 434 | 1.95 |
| Jiangxi | 2353 | 0.30 | Ningxia | 315 | 1.43 |
| Liaoning | 2195 | 0.38 | Qinghai | 298 | 0.25 |
| Fujian | 2129 | 1.38 | Xizang | 172 | 2.09 |
| Shanxi | 1965 | 0.75 |  |  |  |

**Data of figure 3**

| year | Price of urban HR(yuan/person) | Price of rural HR(yuan/person) | National HR Price(yuan/person) |
| --- | --- | --- | --- |
| 1995 | 6209 | 620 | 2183 |
| 1996 | 7086 | 782 | 2604 |
| 1997 | 7529 | 921 | 2888 |
| 1998 | 7819 | 973 | 3068 |
| 1999 | 8406 | 1056 | 3363 |
| 2000 | 8968 | 1160 | 3668 |
| 2001 | 9731 | 1262 | 4068 |
| 2002 | 11456 | 1366 | 4830 |
| 2003 | 12800 | 1486 | 5510 |
| 2004 | 14226 | 1609 | 6246 |
| 2005 | 15440 | 1893 | 7045 |
| 2006 | 17246 | 2218 | 8157 |
| 2007 | 20049 | 2572 | 9754 |
| 2008 | 21963 | 3003 | 11058 |
| 2009 | 23972 | 3343 | 12408 |
| 2010 | 26469 | 3939 | 14208 |
| 2011 | 29644 | 4803 | 16477 |
| 2012 | 33259 | 5591 | 18974 |
| 2013 | 31771 | 5937 | 18770 |
| 2014 | 34183 | 6770 | 20719 |
| 2015 | 36902 | 7495 | 22838 |
| 2016 | 39555 | 8187 | 24933 |
| 2017 | 42532 | 9013 | 27344 |
| 2018 | 45556 | 9898 | 29853 |
| 2019 | 49959 | 11463 | 32120 |
| 2020 | 51438 | 12351 | 33707 |

**Data of figure 4**

| **Province** | **Mean values of the quality adjustment  index of HR** | **Province** | **Mean values of the quality adjustment  index of HR** |
| --- | --- | --- | --- |
| Chongqing | 2.0006 | Qinghai | 1.8107 |
| Beijing | 1.9995 | Ningxia | 1.8082 |
| Jiangsu | 1.9836 | Hunan | 1.8013 |
| Shanxi | 1.9726 | Xijiang | 1.7997 |
| Shanghai | 1.9242 | Shanxi | 1.7836 |
| Guizhou | 1.9025 | Hainan | 1.7732 |
| Zhejiang | 1.9 | Xizang | 1.7647 |
| Tianjin | 1.8837 | Fujian | 1.7643 |
| Henan | 1.8568 | Heilongjiang | 1.7387 |
| Guangdong | 1.8512 | Jiangxi | 1.737 |
| Gansu | 1.845 | Jinlin | 1.7369 |
| Sichuan | 1.8414 | Liaoning | 1.734 |
| Hubei | 1.8379 | Yunnan | 1.7137 |
| Anhui | 1.8332 | Hebei | 1.7077 |
| Shandong | 1.8202 | Guangxi | 1.6981 |
| Neimenggu | 1.811 |  |  |

**Data of figure 5**

| Province | Average nominal  HR prices | Average actual  HR prices | Province | Average nominal  HR prices | Average actual  HR prices |
| --- | --- | --- | --- | --- | --- |
| Shanghai | 37518 | 19091 | Jiangxi | 11807 | 6363 |
| Beijing | 34490 | 16521 | Shanxi | 11580 | 5472 |
| Guangdong | 23403 | 12694 | Hainan | 11569 | 6055 |
| Tianjin | 21641 | 10695 | Heilongjiang | 11569 | 6319 |
| Jiangsu | 18258 | 8606 | Hunan | 11405 | 5779 |
| Zhejiang | 17715 | 9147 | Qinghai | 10782 | 5337 |
| Fujian | 15596 | 8674 | Hubei | 10497 | 5354 |
| Liaoning | 14840 | 8091 | Guangxi | 10342 | 5901 |
| Neimenggu | 13969 | 7216 | Anhui | 10259 | 5183 |
| Shanxi | 13503 | 7071 | Sichuan | 9809 | 4915 |
| Shandong | 13420 | 6809 | Guizhou | 9002 | 4476 |
| Chongqing | 13262 | 6341 | Henan | 8724 | 4215 |
| Xijiang | 12629 | 6675 | Gansu | 8336 | 4044 |
| Hebei | 12314 | 6689 | Xizang | 8244 | 4300 |
| Jinlin | 12242 | 6782 | Yunnan | 7914 | 4256 |
| Ningxia | 12045 | 6023 |  |  |  |
