# Supplementary material for: Quality adjustment and analysis of human resource prices in China: Based on a hedonic price model
Source: PLoS One. 2024 Apr 2;19(4):e0297352. doi: 10.1371/journal.pone.0297352 (PMC10986967; doi:10.1371/journal.pone.0297352)
Supplement: Attachment — Submitted filename: Response to Reviewers.docx [file pone.0297352.s002.docx]

**Response to Reviewers**

**Reviewer #1：**

1. **Describe the challenges and novelty of this research in the abstract and introduction.**

Reply:

**New challenges and novelty in the abstract:**

Firstly, with the Marx's labor value theory and Adam Smith's theory as the theoretical basis, the System of National Economic Accounting 2008 (SNA2008) and the System of Social and Demographic Statistics (SSDS) as the accounting criteria, the price level of human resources in China is calculated. Secondly, establishing the human resource quality index system to measure the human resource quality adjustment index. Third, the Hedonic method was used to adjust the human resource price of 31 provinces (autonomous regions and municipalities directly under the Central Government) in China, and the "pure price" of human resources was obtained without quality factors. Lastly, comparing the price of human resources before and after quality adjustment.

**New challenges and novelty in the introduction:**

The innovations of this paper are in following: First, constructing the human resources quality index system. Second, measuring the human resources quality adjustment index. Third, estimating the "pure price" of human resources after quality adjustment. This paper focuses on China's human resources and calculates the price of human resources in China from 1995 to 2020.

2. **There is no indication that the** **novelty of the paper has been validated by the author.**

Reply:

Novelty1: First, constructing the human resources quality index system.

Verification in the paper: In Section 4.1, the Index System to Measure Human Resource Quality which is showed in Table 2.

Novelty2: Second, measuring the human resources quality adjustment index.

Verification of the paper: In part 4.2.1 and Part 4.2.2, there are respectively "Principle of the Hedonic Price Method" and "Estimation Procedure of the HR Quality Adjustment Index".

Novelty3: Third, estimating the "pure price" of human resources after quality adjustment.

Verification of the paper: In part 5 of the essay, Comparison of Human Resource Prices in Different Provinces, Ranking Changes in Nominal and Actual Human Resource Prices by Province

3. **This paper does not cite all the statistics that were used in the study.**

Reply:

All statistics cited have been used. They are shown in Table 1- Table 5 and Figure 1- Figure 5.

The data used in the table are HR prices in 31 provinces of China (part of years), the average nominal and actual HR prices in 31 provinces.

The data used in the figure are Amount of HR in China from 1995 to 2020, Average amount of HR and average growth rates in China's 31 provinces, National and suburban and rural HR prices from 1995 to 2020, Mean values of the quality adjustment index of HR in 31 provinces, Average nominal and actual HR prices of 31 provinces from 1995 to 2020.

4. **As far as the use of mathematical symbols is concerned, there are no adequate explanations in the text.**

Reply:

I have explained the mathematical notation adequately. Table 2 shows the code of each index in the human resource quality index system. In Part 4.2.1, the symbolic description of formula (6) - (10) is added.

5. **There is no adequate mathematical explanation to support the claim.**

Reply:

Formula (6) - (10) is the mathematical explanation of the human resource quality adjustment index, and formula (15) - (22) is the specific calculation process of the human resource quality adjustment index.

**6.Add a literature review section. The author is advised to include recent research that addresses a similar issue. A summary table of existing studies with strengths and weaknesses should also be presented by the author. The literature from 2021 and 2022 should be included along with more recent studies in the paper to strengthen the literature review section.**

Reply:

The latest literature review is added, such as those from 2021, 2022 and 2023. In part 2.1, 2.2, 2.3, 2.4 and 2.5, respectively.

The advantages and disadvantages of the existing literature are also summarized. At the end of Part 2.5, the content is as follows:

The existing literature provides sufficient theoretical basis for the study of human resource price, which can be measured by the wage level of labor force. However, the existing research on the quality of human resources is limited. Firstly, the construction of human resource quality index system is not comprehensive. Secondly, there is no research on the quality adjustment of human resource price.

6. To interpret the results of the analysis, the author should consider the following papers.

Reply:

I have carefully read the paper for reference. Conclusions of analysis are further explained in 6.1.Research Conclusions.

**Reviewer #2:**

1. **The communication of this article is weak, a throughout proofreading is needed.**

Reply:

The article has been thoroughly proofread for the language.

2. **Add contribution of this article in the end of introduction section.**

Reply:

The article has added some innovative points at the end of the introduction. The contents are as follows:

The innovations of this paper are in following: First, constructing the human resources quality index system. Second, measuring the human resources quality adjustment index. Third, estimating the "pure price" of human resources after quality adjustment. This paper focuses on China's human resources and calculates the price of human resources in China from 1995 to 2020.

3. **Use proper statistical software for graphs.**

Reply:

Statistical software SPSS is used in this paper, which is explained in the upper text of Table 3.
